# Common data elements for secondary use of electronic health record data for clinical trial execution and serious adverse event reporting

**DOI:** 10.1186/s12874-016-0259-3

**Published:** 2016-11-22

**Authors:** Philipp Bruland, Mark McGilchrist, Eric Zapletal, Dionisio Acosta, Johann Proeve, Scott Askin, Thomas Ganslandt, Justin Doods, Martin Dugas

**Affiliations:** 1 Institute of Medical Informatics, University of Münster, Münster, 48149 Germany; 2 University of Dundee, Dundee, DD2 4BF UK; 3 Département d’Informatique Hospitalière, AP-HP, Hôpital Européen Georges Pompidou, Paris, 75015 France; 4 CHIME, Institute of Health Informatics, University College London, London, NW1 2DA UK; 5 Previously Bayer Healthcare, Building K9, Leverkusen, 51368 Germany; 6 Novartis Pharma AG, Basel, 4002 Switzerland; 7 Chair of Medical Informatics, University of Erlangen/Nuremberg, Erlangen, 91054 Germany

**Keywords:** Clinical trials, Common data elements, Data quality, Electronic health records, Metadata, Secondary use

## Abstract

**Background:**

Data capture is one of the most expensive phases during the conduct of a clinical trial and the increasing use of electronic health records (EHR) offers significant savings to clinical research. To facilitate these secondary uses of routinely collected patient data, it is beneficial to know what data elements are captured in clinical trials. Therefore our aim here is to determine the most commonly used data elements in clinical trials and their availability in hospital EHR systems.

**Methods:**

Case report forms for 23 clinical trials in differing disease areas were analyzed. Through an iterative and consensus-based process of medical informatics professionals from academia and trial experts from the European pharmaceutical industry, data elements were compiled for all disease areas and with special focus on the reporting of adverse events. Afterwards, data elements were identified and statistics acquired from hospital sites providing data to the EHR4CR project.

**Results:**

The analysis identified 133 unique data elements. Fifty elements were congruent with a published data inventory for patient recruitment and 83 new elements were identified for clinical trial execution, including adverse event reporting. Demographic and laboratory elements lead the list of available elements in hospitals EHR systems. For the reporting of serious adverse events only very few elements could be identified in the patient records.

**Conclusions:**

Common data elements in clinical trials have been identified and their availability in hospital systems elucidated. Several elements, often those related to reimbursement, are frequently available whereas more specialized elements are ranked at the bottom of the data inventory list. Hospitals that want to obtain the benefits of reusing data for research from their EHR are now able to prioritize their efforts based on this common data element list.

**Electronic supplementary material:**

The online version of this article (doi:10.1186/s12874-016-0259-3) contains supplementary material, which is available to authorized users.

## Background

Data collection is one of the most expensive processes during the conduct of clinical trials. Over the last decade the number of clinical trials and the size of trials have steadily increased [1]. Likewise, the number and complexity of case report forms (CRFs) capturing the data for trial subjects grew as well. From a hospital perspective, the use of electronic health record (EHR) systems and consequently the number of patients having at least a basic electronic medical record has experienced a significant and steady growth [2]. The transition from paper-based to electronic documentation has resulted in clinicians spending around 25–30% of their time on electronic documentation tasks [3, 4].

Recent research has shown that a certain amount of EHR data elements are available and suitable for different research purposes [5–9]. Nevertheless, it is important to note that the provenance, availability, degree of standardization and structure of the EHR data plays a major role in its re-use for clinical research purpose [10–12].

Currently, the exchange of routinely collected data between EHR systems and clinical research databases is not fully automated and requires human intervention. This manual step is time-consuming, error-prone and also demotivating [5]. Transferring data electronically from an EHR source into an electronic data capture (EDC) system in a systematic, auditable and unambiguous manner provides several advantages, avoiding the detrimental effects of repeated data entry, decreasing documentation time and improved data quality and cost-effectiveness [5, 7, 13, 14].

On-site data monitoring is an expensive process for pharmaceutical companies as well. Monitors have to visit all sites to perform the so-called’Source Data Verification’ (SDV) by comparing source materials at the sites with data that has been entered into the trial database, e.g. an Electronic Data Capture (EDC) system. This tedious, time-consuming and expensive process might be optimized through a connection between EHR systems at the sites and the EDC system. If such a link is established and presumed data are validated, SDV could likely be reduced or even be eliminated. Time-consuming site visits would be reduced to a minimum and the monitors could focus on other aspects of the clinical trial conduct.

The Electronic Health Records for Clinical Research (EHR4CR) project [15], which is funded by the Innovative Medicines Initiative (IMI) has investigated these potential incentives and benefits [16]. The project is a public-private-partnership consisting of 34 partners from European pharmaceutical industry and academic institutions. Clinical partners were from France, Germany, Poland, Switzerland, and the United Kingdom. The participating companies from the European Federation of Pharmaceutical Industries and Associations (EFPIA) were: AMGEN, AstraZeneca, Bayer Health Care, F. Hoffmann-La Roche Ltd, GlaxoSmithKline, Johnson & Johnson, Lilly, MERCK KGaA, Novartis Pharma AG and Sanofi-Aventis.

The project’s aim was to develop methods and a software platform as well as an accompanying business model to support clinical trials based on routinely collected data from EHR systems. Addressed scenarios included ‘protocol feasibility’, ‘patient identification and recruitment’, ‘clinical trial execution’ (CTE) and ‘serious adverse event reporting’ (SAE). Disease areas which the project focused on were diabetes, cardiovascular, infectious, oncology, neurology and respiratory diseases. In addition to establishing the benefits associated with the first two scenarios, the ‘business model’ workgroup also showed substantial potential savings in the scenario of ‘clinical trial execution’ [17].

The EHR4CR net benefits are obtained by offsetting these savings against the expenditure for setting-up the infrastructure to allow the re-use of routinely collected clinical data. Suitable data elements need to be identified, new structures for documentation procedures might have to be established, and dedicated exports from the source EHR to the research database have to be maintained. In order to control and reduce these operational costs, the best approach is to focus on the most frequently used data elements of clinical trials.

The ability to pre-specify common data elements (CDEs) would greatly improve the setup process of electronic databases and simplify the exchange of medical data between different systems, i.e. EHR and EDC systems. Subsequent analyses are then accelerated due to fewer data transformations from different sources, and enhance the comparability of outcomes. Additionally, CDEs might help to reduce the number of, and focus on, relevant data elements that should be captured across all and in certain therapeutic areas. These effects should be especially favorable in the context of multicenter trials that could benefit from a common data model. Several initiatives and research groups have tackled this issue and defined common data elements for different therapeutic areas [18–22]. Common data elements are defined as metadata information that is of interest or relevance in a specific research domain. As part of the EHR4CR project, common data elements for the scenarios of protocol feasibility and patient identification and recruitment have previously been determined by Doods et al. [23, 24].

In order to re-use CDEs for clinical trials, they must firstly be available for documentation in the EHR information systems and secondly must be actively used to contain patient data. Several research groups have examined the presence of clinical trial data elements within existing EHR systems and found a broad range of coverage between 13 to 70% [5–9]. However, these results were only investigated for one clinical trial [5–7, 9] and an analysis of the frequency of documentation has so far only been performed in very few cases (e.g. Botsis et al. [25]).

Nevertheless, it remains unclear what kind of data elements are most commonly required for the documentation of clinical trials and whether those elements are available and also captured across EHR systems.

### Objective

Work Package 7 (Pilots) of EHR4CR developed an inventory of relevant data elements as an important cornerstone for the development of a system facilitating the secondary use of routinely collected data for the subject documentation in clinical trial. An inventory also supports calculations for business modeling to estimate whether this approach is economically feasible. The aim of the present research is to determine what data elements are the most frequently used in clinical trial execution. An additional focus was on data elements supporting SAE reporting. The fundamental question is whether those data elements are covered by European EHR systems and how frequently they have been captured.

## Methods

An iterative consensus-driven approach was chosen for creating the inventory of CDEs for CTE and SAE reporting as well as their completeness of documentation.

### Material

CRFs from clinical trials were collected from all participating EFPIA companies within the EHR4CR project to perform the analysis. Criteria for CRF selection were at least one comprehensive clinical trial including over 200 study locations and a four-digit planned patient enrollment number. According to the Good Clinical Practice guideline, CRFs are understood as printed, optical or electronic documents designed to record all of the protocol-required information to be reported to the sponsor on each trial subject [26]. A CRF consists of several forms, each one with a different purpose/domain (e.g. demographics, vital signs, adverse events, and also multiple instances of, sometimes unscheduled, follow-up forms). We analyzed 23 trials covering the following disease areas as listed in table 1: cardiovascular, diabetes, infectious, neurology, oncology, psychiatric and respiratory. The reviewed clinical trial forms ranged between 22 and 164 and contained in sum 1086 forms. We used all forms of a CRF for the analyses, also repeating and unscheduled ones. The most comprehensive clinical trial amounted to a total of 3581 data elements.

**Table 1:** Numbers of clinical trials and forms that were collected per disease area

Disease area	No. of trials	Forms
Cardiovascular	3	158
Diabetes	3	172
Infectious	2	60
Neuroscience	1	64
Oncology	3	192
Psychiatric	1	69
Respiratory	10	371
Sum	23	1086

The CRFs were in different computer processable formats such as XML-based Operational Data Model from the Clinical Data Interchange Standards Consortium (CDISC) or Excel spreadsheet files. Apart from CDISC Operational Data Model, all files were confirmed as having different structures.

For the availability and completeness evaluation we analyzed data exports from seven hospital sites for the data inventory. Some sites provided data from hospital-wide EHR systems while others only data from subsystems or data warehouses, for instance specialized systems for breast cancer or diabetes. For one site data was only available from in-patient cases.

### Methodology

After collecting the trial CRFs, medical informatics professionals from academia and trial experts from EFPIA in the EHR4CR project were involved in the consensus-driven process for creating the data inventory. Data exports were performed by different university hospitals across Europe to assess the availability of these distinct elements and their frequency of documentation.

**Fig. 1:**
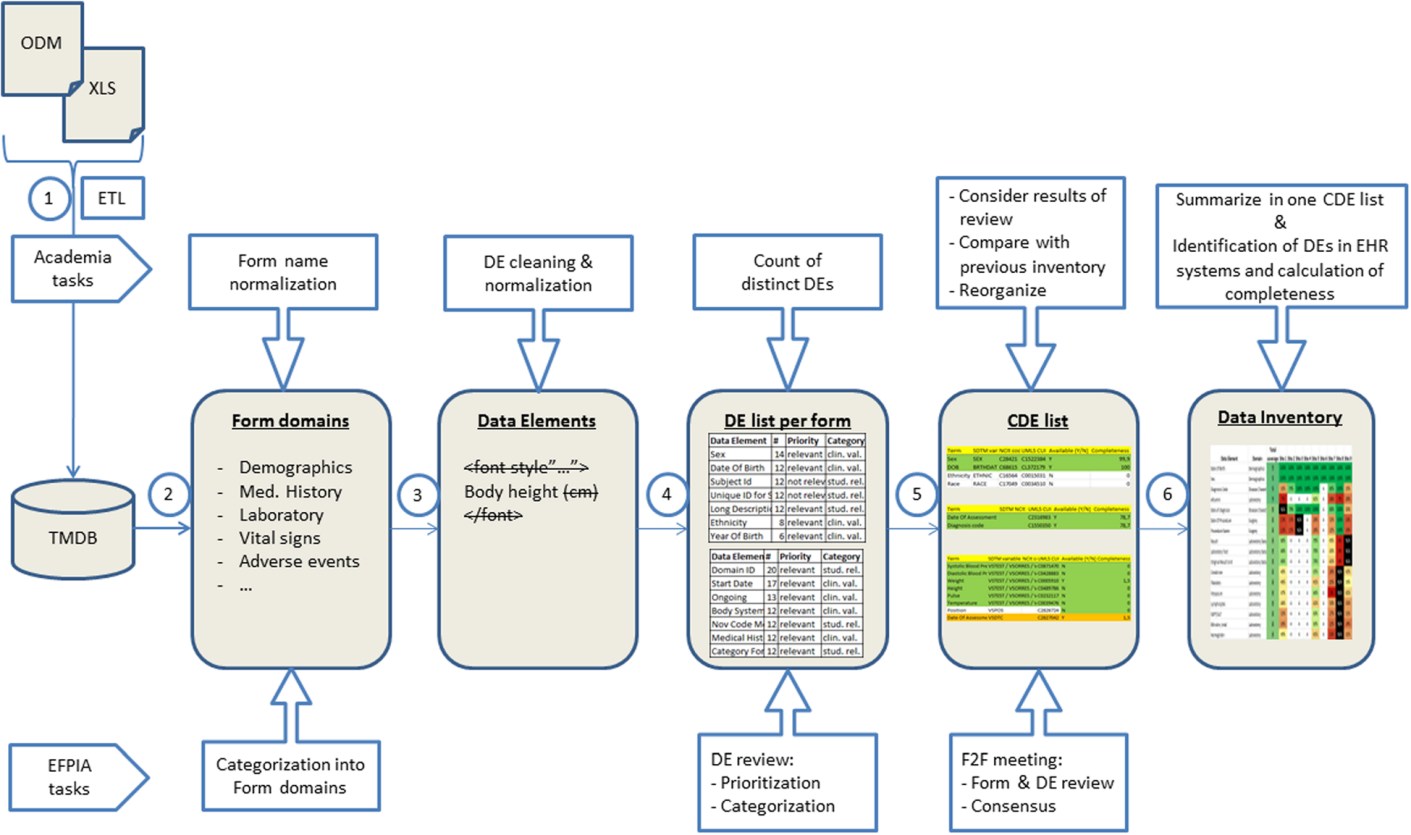
Process steps to create the data inventory for common data elements in clinical trials and their frequency of documentation in European EHR systems. First, clinical trials were collected and imported into the trial master database (TMDB). Then forms were categorized into domains, and all data elements were normalized. Top data elements per form domain were determined. Top form domains were reviewed by EFPIA partners. Afterwards, at a face-to-face meeting all elements were jointly discussed with academia and pharmaceutical partners. Finally, clinical sites identified element presence and performed data exports to determine the frequency of documentation

Figure 1 illustrates the process and involved parties who conducted the analyses for CTE and SAE reporting.

**Fig. 2:**
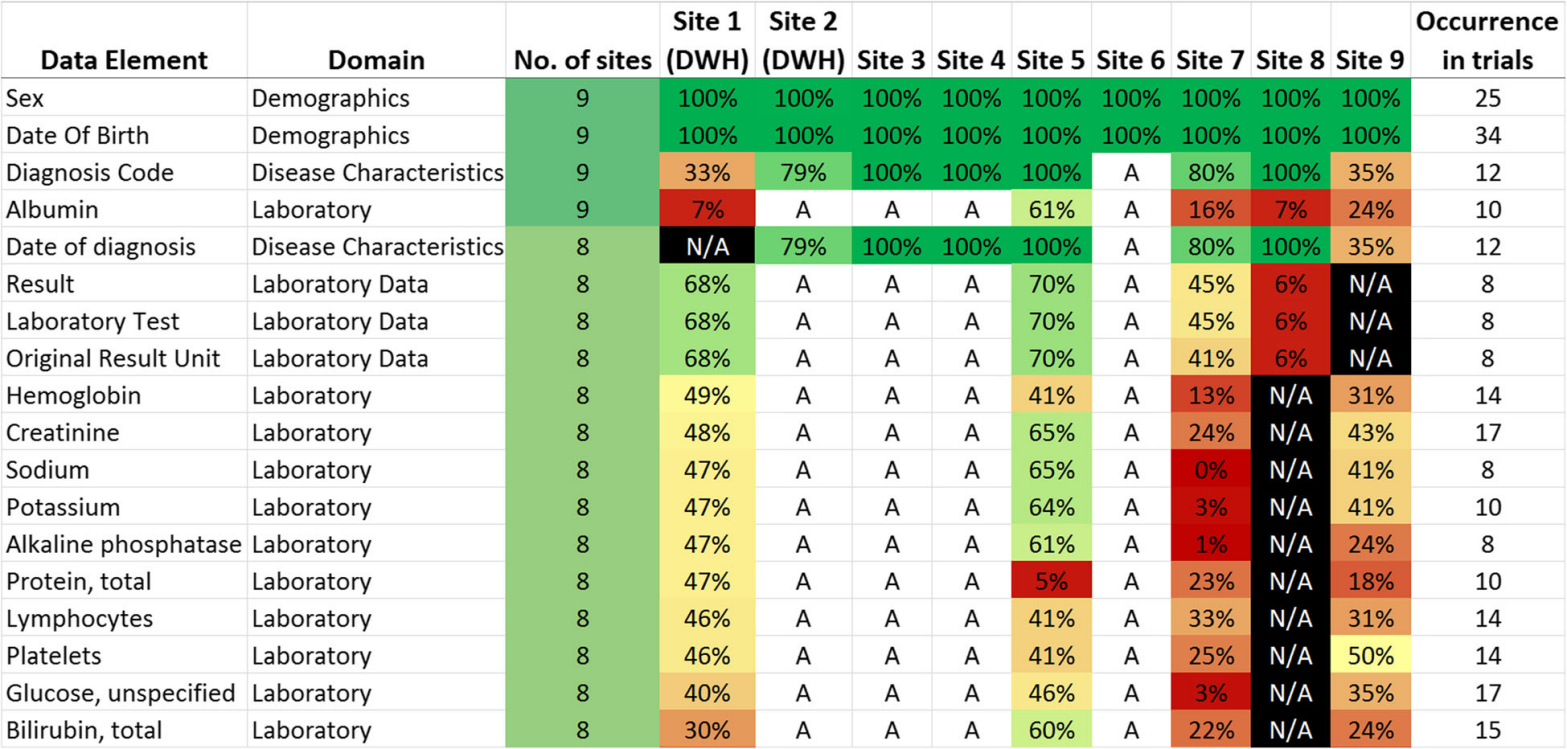
Extract from the entire data inventory for clinical trial execution and SAE reporting. On the left-hand side the data elements and their form domains are listed followed by the number of sites in which they occur and the sites availability. Site 1 and 2 used their data warehouse (DWH) for element identification and exports

**Fig. 3:**
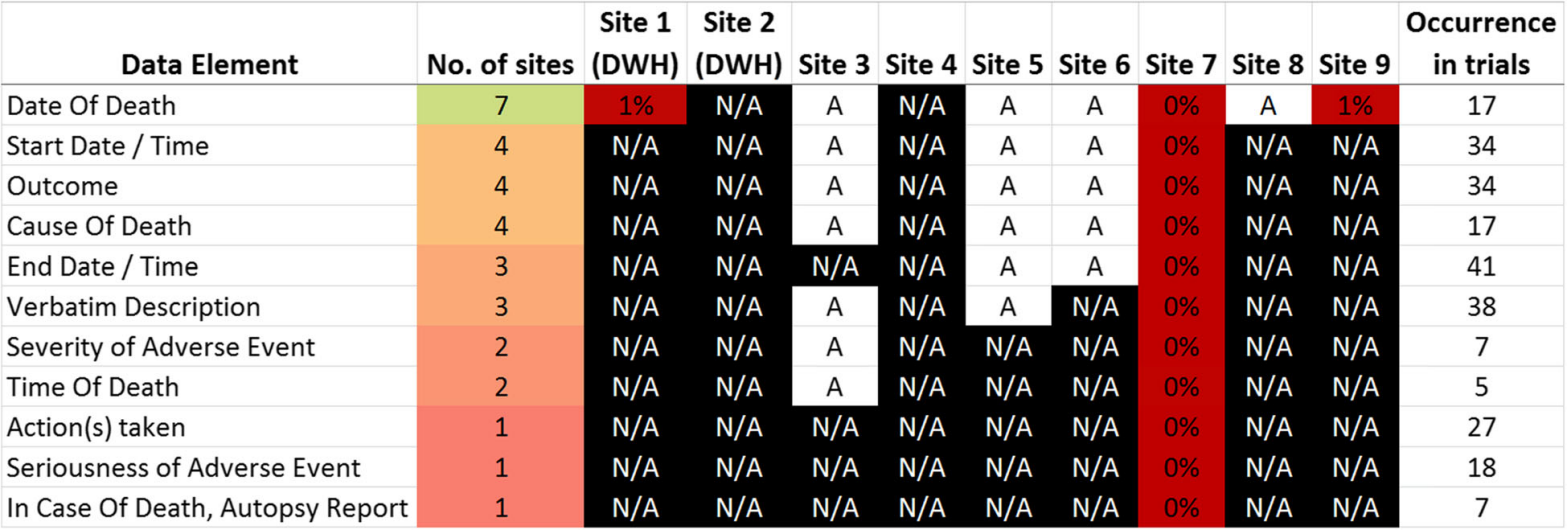
Common data elements for the reporting of SAEs. More than half of the data exporting sites have no SAE documentation available in their EHRs. This refers to the items ‘seriousness’, ‘action taken’, and ‘autopsy report in case of death’. Some sites have few data elements available but unclear whether data values are present apart from one where no data has been captured so far

The following steps were performed:

1. Import: First, all collected CRFs were transformed and loaded into the TMDB (Trial Master Database) using the ETL-tool ‘Talend Open Studio for Data Integration’ [27]. The TMDB is used to gather all differently structured clinical trial CRFs together within one structured database. Meta-information for each trial is included concerning the disease area, planned patient enrollment numbers and participating sites. All CRFs and the associated data elements were imported into the TMDB.
2. Form categorization: To determine a top list of form, they need to be categorized by a domain, which was allocated by trial experts from EFPIA, each focusing on the trials provided by their company. Domains are understood as topic-specific classes (i.e. ‘medical history’, ‘vital signs’ or ‘concomitant medications’) that deliver additional contextual information to sites when exporting information on data elements they hold. Where possible, we assigned preferably the domains of CDISC’s SDTM (Study Data Tabulation Model) [28], which is used for the definition and transmission of trial data. For instance, the form name ‘Coagulation Panel’ was allocated with domain ‘Laboratory test results’ or ‘LB’ in SDTM.
3. Data element normalization: During form categorization, data element names were normalized using phonetic and word similarity measures such as Levenshtein distance [29], Jaro-Winkler distance [30] and Metaphone [31]. This was a relevant process for the determination of common data elements. Special characters and HTML tags as well as style sheet information were removed using regular expressions. Measurement units were also removed since a conversion of values is feasible. If CDISC variable names were provided for an element, these names were additionally used for the normalization of element names. Finally, a manual review by trial experts was conducted to validate this part of the process and determine results for those cases which could not be matched.
4. Data element review: Forms are used to collect data in clinical trials, therefore for the list of common data elements we chose to first rank the form domains by the frequency with which they appear in CRFs. We removed form domains that only appeared once. Then we calculated the frequency of each unique data element within each form domain. As a weighting factor we used the planned patient enrollment numbers we had available for each trial. There is an expectation that certain data elements will be found in certain form domains and not in others. Data elements within each form domain were independently reviewed by two EFPIA trial experts concerning an element’s relevance to the associated form domain. Duplicates were detected and CDISC variable names were added where possible. If data elements relevant to the domain were missing, the trial experts added them to the particular form domain. Distinctions between clinical parameters or administrative values (e.g. sequence numbers, subject or site identifiers) were also made to state whether data elements are expected to be re-used from EHR systems. Due to the association between form domains and data elements, the frequency list for distinct CTE and SAE elements could be created.

5. Consensus meeting: As a last step, we refined the frequency lists in a face-to-face consensus meeting at which academic as well as pharmaceutical partners participated. The goal of this meeting was firstly to discuss and vote about the allocated form domains, data element names and semantic codes. It was stated whether a form domain should be kept in the overall list, whether an element should be removed in a form domain, and, if so, whether the naming was correct, whether it was a duplicate and finally which category (clinical parameters or administrative values) it belonged to. For instance, most elements that were stated as irrelevant were removed. After this consensus-driven step, the list was compared with the previous data inventory of ‘patient identification and recruitment’ to determine which elements had already been examined at the data provider sites. Some sites’ health information systems or data warehouses contained semantically annotated data elements. To support the discovery, mapping and data export process, semantic codes were assigned to the elements in the list of the common data elements. Data elements of many CRFs already contained SDTM codes, so automated mappings to codes of the Unified Medical Language System (UMLS) were performed. Elements without SDTM codes were manually annotated by a medical informatics professional and a medical expert. SNOMED-CT (Systematized Nomenclature of Medicine – Clinical Terms) codes were additionally added through a semi-automated identification process using the available mapping in the UMLS database.

6. Presentation of frequency list: Finally, the CDE list was distributed to the participating European university hospitals within the EHR4CR consortium. Data elements were located within structured documentation of their local data warehouses (previously populated with data from EHR systems), EHR systems or specialized subsystems and the frequency of occurrence was assessed, where possible. Sites with semantically annotated data sources programmatically identified matching entries between the CDE list and their source system based on identical results of code system codes. Frequency was calculated using the number of entered values which have been documented in the year 2013 divided by the total number of patients in 2013. To address privacy concerns and to obtain comparable values, relative percentages for a data element were given. For instance, a frequency of 30.4% for ‘Bilirubin, total’ is the result of dividing the number of entered values (9574) by the number of patients in the year 2013 (31493). Where frequencies were not calculated in figs. 2 and 3, a distinction was still made between data elements that were ‘available’ (understood as possibility that the data element can be stored in the EHR system) and ‘not available’ for a given site. A heat map of data element frequency was created to depict the whole data element coverage, determined by the number of available elements. The heat map for SAE elements was reported separately. All analyses were performed using Microsoft Excel.

## Results

### Data inventory

After the consensus meeting 14 form domains remained relevant for potential pre-population in clinical trials. Table 2 shows the final form domains used in clinical trials with SDTM domain abbreviations where available and the frequency of occurrence, number of total forms and data elements that are contained in the respective domain.

**Table 2:** Consensus list of top 14 form domains in order of frequency of occurrence. This list also includes the SDTM domain abbreviation if available and in how many trials the domain is used, the number of total forms and the containing data elements

Form domain	SDTM domain	In no. of trials	No. of forms	No. of data elements
Medical History	MH	23	85	1336
Adverse Event	AE	23	78	2733
Laboratory test results	LB	10	75	1643
Disposition	DS	19	74	653
Vital Signs	VS	23	56	974
Concomitant Medications	CM	22	52	1334
Questionnaire/Patient reported outcome	QS	16	35	1122
Demographics	DM	23	34	371
ECG	EG	12	30	436
Disease Characteristics	(ZC)	12	25	190
Substance Use	SU	14	22	246
Surgery	-	9	20	80
Physical Examination	PE	5	10	33
Tumor response	RS	1	7	27

Medical History, Adverse Events, Laboratory and Disposition are ranked at the top. Apart from Surgery, Physical Examination and Tumor Response all domains were present more than once in a clinical trial. SDTM domains could be identified for all forms apart from ‘Surgery’ and ‘Disease Characteristics’. As shown in table 2 domains like ‘Demographics’, ‘Medical History’, ‘Adverse Events’ and ‘Vital Signs’ are used in all trials; ‘Tumor response’ only in one trial. The number of unique data elements which was the basis of these analyses ranged between 27 in ‘Tumor response’ and 2733 in ‘Adverse Events’.

After the consensus meeting the final data inventory was created which contains 133 data elements that are identified as the most frequently used elements in clinical trials. In a comparison with the previous data inventory for patient identification and recruitment 50 data elements are identical and the remaining 83 elements are new for the execution of clinical trials.

### Availability and frequency in European EHR systems

Figure 2 presents an extract of the most frequently used data elements in clinical trials sorted by the frequency of captured elements in EHR systems. We differentiated between the presence (A=available; N/A=not available) and the frequency of captured data elements. Demographic and reimbursement data was ranked at the top of the list followed by several laboratory test results. The number in the right column indicates the frequency of occurrence in all forms of all trials.

The complete data inventory can be accessed in the additional material [see Additional file 1]. It also contains semantic codes of the UMLS and where possible of SNOMED CT as well as a definition of each data element. In terms of reporting SAEs, fig. 3 shows the most frequently used data elements for the domain of SAEs.

Over half of the data providers reported not having any documentation structure to collect SAE information at all. Three reported that they have at least some elements available but it was unclear whether data has been collected in 2013. Site 7 has a complete electronic documentation for SAEs available within their EHR, but has never been used. Apart from the ‘date of death’, SAE related data has not been collected at all.

The complete inventory of data elements for ‘clinical trial execution’ and ‘serious adverse event reporting’ can be accessed online at: https://www.medical-data-models.org/forms/17994.

## Discussion

Re-use of routinely collected medical data is a promising approach to some of the problems clinical trials currently face. In most projects that attempt to use EHR data for purposes other than patient care, the re-use requires manual mapping between EHR and clinical trial data elements. To keep these efforts to a minimum, it is desirable to know which elements are most commonly used in clinical trials, to what extent such elements are available in EHRs and also to know how frequently such elements are currently documented.

The present research generated a list of common data elements found in clinical trials. It was compiled through an iterative and consensus-based process with medical informatics professionals from academia and trial experts from the European pharmaceutical industry. Through data exports performed at different university hospitals across Europe this list also presents the potential for re-use of EHR data in clinical trials. Different source systems, languages and terminologies underline the complexity of this research.

A large proportion of the data elements are laboratory analytes that are commonly required in clinical trials. These data elements are usually well structured within EHR systems so that these elements could be identified. During this localization process within the systems documentation structure it became apparent that some elements were present in multiple forms within the EHR. So, the origin and purpose had to be clarified also with the aid of the form domains and further semantic annotation. Especially the semantic annotation of data elements is a crucial but also one of the most labor-intensive and tedious tasks to be performed to facilitate the re-use of routinely collected healthcare data. A further issue was that several clinical concepts are only available in unstructured form as free text in the EHR. Most frequently available are demographic elements, captured by all hospitals followed by diagnostic and procedural entities. The remaining data elements are far less often captured, which is expected since the main driver for data modeling in EHR systems is support of regulation, policy and reimbursement rather than clinical practice or research.

Since the CDE analysis did not focus on disease-specific data elements, exports by certain departments were not possible. Analyses for disease specific elements might have resulted in higher frequency values because disease-related elements are more frequently documented in their disease area. Some data elements are common between disease areas, whereas others do not belong to the particular subset. For instance, biopsy results would not exist in the cardiovascular disease area but rather in oncology, and conversely for laboratory results in cardiovascular disease. The site identification of these data elements and their associated data exports was a labor intensive and time consuming process for all participating sites. Conducting these analyses for elements in all disease areas would increase the workload substantially since data exports would need to be performed for each element in each area separately; dependent on how uniquely such elements are captured.

An additional focus of this work was to assess the coverage of data elements for SAE reporting. Not surprisingly, all clinical trials examined contained CRFs for adverse events. Our analysis showed that elements are highly standardized and also related to the SDTM domain of adverse events (AE). Nevertheless, apart from ‘date of death’ SAE elements such as start and end date, outcome, verbatim description, severity and seriousness or action(s) taken were not captured in the EHR system at all. This underlines that clinical practice and trial execution are different conceptual domains with different purposes for data capture.

However, it must be considered that some clinical phenomenon may not be directly collected as a data element but rather derived from different elements or different perspectives. For instance,’cause of death’ might be related to several finer grained data elements, such as biomarkers being completely out of normal range. Although they might be recorded, the ‘cause of death’ reported would be determined by a forensic pathologist, which could be very different from the perspective of a clinician. Hence, it might be worthwhile to investigate further the data structure of information systems, the purpose of data elements and the human rather than technical process of data collection.

For the development of the data inventory we chose to perform this process iteratively. This led us to a modified representation for the element list. In the previous EHR4CR data inventories only the frequency was stated. Instead, we decided to additionally indicate whether a data element is just available regardless of whether its frequency could be stated or not. A frequency of 0% would imply that the data element is present but data were never collected. ‘Available’ gives the information that data might have been captured but could not be assessed.

### Related work

In the EHR4CR project, data inventories for ‘protocol feasibility’ [24] and ‘patient identification and recruitment’ [23] have been performed by Doods et al. There, 75 data elements were identified for feasibility assessment and 150 data elements for patient identification and recruitment. Despite the differing scenarios, a comparison with the current inventory for the execution and SAE reporting in clinical trials has shown that 50 data elements have already been identified and 83 are new data elements.

CDISC, C-Path, NCI-EVS and CFAST had introduced an initiative on ‘Clinical Data Standards’ to create industry-wide common standards for data capture in clinical trials to support the exchange of clinical research and metadata [32]. This initiative defines common data elements for different therapeutic areas. Currently, traumatic brain injury, breast cancer, COPD, diabetes, tuberculosis, etc. are covered. In addition, the CDISC SDTM implementation guideline contains a set of standardized and structured data elements for each form domain. The aim of this initiative is similar to ours concerning the identification of most frequently used data elements for clinical trials. Nevertheless, the focus of our work is different and goes beyond this initiative in terms of determining the availability and quality of data within EHR systems.

Köpcke et al. have analyzed eligibility criteria from 15 clinical trials and determined the presence and completeness within the partners EHR systems [33]. Botsis et al. examined the incompleteness rate of diagnoses in pathology reports resulting in 48.2% (1479 missing of 3068 patients) [25]. Both publications show that re-use of EHR data relies on the availability of (1) data fields and (2) captured patient values.

### Limitations

This research work aimed to build a data inventory for CTE and SAE reporting within the IMI funded EHR4CR project. Therefore, the inventory represents data elements that are important for trials conducted by European pharmaceutical companies as well as showing the availability and frequency within large European university hospitals. The number of clinical trials in this analysis is limited since most trial metadata are not publicly available [34] and EFPIA partners have provided a small number of clinical trials for the analyzed disease areas. So, this research could be treated as a pilot study and as a foundation for a more comprehensive analysis.

Data exports at the sites have been performed on different sources due to different site specific data access policies: two sites queried their clinical data warehouses; four exported data directly from the EHR and because of permission restrictions two sites did not have the possibility to access patient data at all. One site was only able to export data from a dedicated system for one clinic. The time period for data analyses was the year 2013. At one site data for 2013 was not available in the data warehouse, so, they chose 2012 for their queries. Another site was only able to take the first half of the year for exports. It includes in-patients as well as out-patients (apart from one site) for all medical disciplines available in the source systems.

The data exports of this research represent only data of nine university hospitals across Europe. The generalizability of this approach relies on several aspects: First of all the adoption of EHR systems is a crucial indicator whether data could be made electronically available. The degree of digitalization is also a key factor. Although an electronic system is available it is not necessarily being used. Often, paper is still used in parallel. Last but not least, the degree of structuredness plays an essential role as to whether data is eligible for re-use.

Data elements in clinical trials are highly standardized and EHR documentation forms often contain unstructured information as free text, notwithstanding, initiatives or tools like openEHR [35] or Clinical Element Model [36] aim at defining standardized data elements for clinical documentation. Even though projects like SHARPn [37] or cloud4health [38] use natural language processing techniques for extracting relevant information from free text documents, the EHR4CR project did not focus on this approach.

### Further research

Several pharmaceutical companies have private data standards catalogs of forms which are used to create CRFs for clinical trials. Such catalogues ensure that a clinical trial doesn’t have to start from scratch when setting up a new trial. In this regard, the pharmaceutical companies may benefit from each other when using a standardized catalog of data elements in certain areas together.

A further step for the EHR4CR project is the introduction of the CTE platform within the overall infrastructure. Consequently, the approach of electronic data exchange from patient care to clinical research has to be evaluated. It is also worthwhile to examine whether the application of the EHR4CR platform generates the desired advantages and savings for both sides: time saving and error-reductions for study nurses/sites and reduced SDV as well as more rapidly conducted data documentation for the industry or academic researchers. Reduced expenditure of time for SDV and data collection enables to focus more on other value added activities such as training and recruitment.

Similar to this work, most of the researches focus on the availability and quality of routinely collected patient data. However, it remains unclear whether the available common data elements are exactly the required elements for a clinical trial and whether the procedure of documentation satisfies the needs. For instance, the timeliness and the relationship between data elements play essential roles. The identification of suitable EHR data elements have been performed in all conscience, but the context of documentation remains at times unknown and is also controversially discussed in the literature [10, 39].

Further investigations concerning the data quality and the purpose of documentation are essential to ensure that correct data elements are selected for the secondary use of patient care data, in particular for a clinical trial execution, and that their contribution toward monitoring of therapeutic efficacy, patient-safety and cost-effectiveness can be clearly assessed.

## Conclusions

Common data elements in clinical trials have been identified and their availability in hospital systems elucidated. Several elements, often those related to reimbursement, are frequently available whereas more specialized elements are ranked at the bottom of the data inventory list. Hospitals that want to obtain the benefits of reusing data for research from their EHR are now able to prioritize their efforts based on this common data element list.

## Abbreviations

CDE: Common Data Element; CDISC: Clinical Data Interchange Standards Consortium; CRF: Case Report Form; CTE: Clinical Trial Execution; EDC: Electronic Data Capture; EFPIA: European Federation of Pharmaceutical Industries and Associations; EHR: Electronic Health Record; EHR4CR: Electronic Health Record for Clinical Research; IMI: Innovative Medicines Initiative; SAE: Serious Adverse Event; SDTM: Study Data Tabulation Model; SDV: Source Data Verification; TMDB: Trial Master Database; UMLS (Unified Medical Language System)

## Additional files

Additional file 1: Title of data: CTE & SAE Data Inventory. Description of data: List of common data elements in clinical trials with domain, availability/completeness, occurrence in trials, semantic codes and definition. (XLSX 34 kb)
